# Positive Psychology in Times of Pandemic—Time Perspective as a Moderator of the Relationship between Resilience and Meaning in Life

**DOI:** 10.3390/ijerph182413340

**Published:** 2021-12-18

**Authors:** Agnieszka Lasota, Justyna Mróz

**Affiliations:** 1 Institute of Psychology, Pedagogical University of Krakow, 30-084 Krakow, Poland; 2 Department of Psychology, Jan Kochanowski University, 25-029 Kielce, Poland

**Keywords:** time perspectives, resilience, meaning in life, MIL

## Abstract

Resilience and meaning in life are significant indicators of psychological well-being and health, which are particularly important in the context of the COVID-19 pandemic. Therefore, they have been explored by a growing number of scientists. There has been a research gap, however, that fails to show that time perspectives also have a significant impact on the perception and building of different life aspects. The current study investigated the associations between resilience, time perspectives and meaning in life and examined the moderating role of time perspective in the relationship between resilience and meaning in life. Methods: Participants of this cross-sectional study were 363 adults aged 18-70. Resilience Measurement Scale (SPP-25), the Zimbardo Time Perspective Inventory (ZTPI), and the Purpose in Life Questionnaire (PIL) were used. Results: The findings confirmed a positive relationship between resilience, meaning in life, and positive time perspectives (Present Hedonistic and Future) and a negative link with Past Negative and Present Fatalistic perspectives. The linear regression analyses showed that Past Negative and Past Positive perspectives significantly moderated the relationship between resilience and meaning in life. The moderating effect was also confirmed in the case of past time perspectives only. Conclusions: The findings indicate the relevance of positive resources, such as resilience and positive perception of the past, in keeping the meaning in life. Understanding the effect of psychological strengths in the context of the pandemic time can be a key to providing intervention and therapeutic services fostering mental health and well-being.

## 1. Introduction

In the context of the COVID-19 pandemic, psychological resources, such as resilience of positive perception of the past or future, and finding meaning in life may be extremely important for the ability to adjust to the present situation and for maintaining health and psychological well-being. Positive psychology indicates resilience as a relatively stable personality trait, described as an optimistic approach to focus on difficult situations, openness to new experiences, coping skills, tolerance of negative emotions, determination in action, and tolerance of failures [1]. Resilience leads to better coping with difficulties [2], better quality of life [3], and psychological well-being. Resilience is a natural ability to adapt to stressful events [4] and presents itself as one of the positive traits that can also be relevant to meaning in life [5]. Meaning in life (MIL) is a personal existential strength to face adversity and daily challenges. According to Frankl [6], a resilient person is able to keep the sense of meaning in life despite adversities or life threats.

Earlier research has confirmed various relationships between resilience and MIL. Some studies indicate that meaning in life promotes resilience [7], while others show that resilience may be a predictor of MIL [5]. In our study, we focused on resilience as a predictor of MIL to develop a greater understanding of this relationship and to identify possible mediating variables. The potential association between resilience and MIL has been directly examined only in a few studies to date (e.g., [5]). Therefore, further research may make a valuable contribution to the existing body of knowledge on the subject. Additionally, earlier studies have not focused on the moderating role of different variables in the relationship between resilience and MIL to gain a better understanding of this relationship. Some researchers indicate that temporal references are activated during the coping process, such as consideration of past time [8] or present time [9]. Additionally, previous research showed that temporal orientation is linked to resilience [10,11] and some factors defining resilience [1], such as optimism [12], coping [13], or sense of humor [14]. Previous studies also found a relationship between time perspective and meaning in life [15,16]. Therefore, we wanted to explore the moderating effect of individual differences in time perspective on the relationship between resilience and MIL. The study adds to the body of knowledge on whether orientation toward past, present, or future is relevant to the association between resilience and MIL. Moreover, it can be a starting point for constructing therapeutic or preventive programs in such a way as to foster temporal orientations that enhance positive resources. 

### 1.1. Resilience

Resilience is a complex multidimensional construct that is of great importance for the proper functioning of humans and their health, well-being, and quality of life [3]. Since there is no consensus definition of resilience, it can be defined and measured using a variety of methods. In the literature, resilience is defined as a personality trait that enables individuals to adapt to the circumstances they encounter [2]. It is the ability to bounce back from negative emotional experiences and adapt to the changing demands of stressful experiences [17]. In a similar way, Ogińska-Bulik and Juczyński [1] define resilience as the ability to flexibly respond to changing circumstances, including stress, conflict, or uncertainty. It is also treated as a dynamic psychological process, which includes positive adaptation over time despite adversity [18]. Resilience is the ability to withstand or recover quickly from difficult conditions [19]. It can also be considered as an outcome following adversity [20]. A question still remains open as to whether resilience changes over a lifetime or is stable regardless of the circumstances. The vast majority of researchers believe that resilience is a relatively permanent disposition that determines the process of flexible adaptation to the constantly changing life requirements [4,17]. 

Resilience is defined as the ability to overcome stress and adversity and to maintain proper mental health. It is the key to overcoming difficulties and adversities everyone has to face at some point in life [21]. Highly resilient individuals look for bright sides of the circumstances they have to face, use their strengths to cope with adversities, and look for possibilities to protect their mental health, as opposed to individuals having a lower level of resilience who focus on their weaknesses and easily surrender to a stressful situation [22]. 

Many researchers have posited that resilience should not be considered solely in the context of facing trauma and that it is highly significant in daily life, without trauma but not free of adversity, which is related to the sense of coherence [23]. Resilience displays positive correlations with protective mechanisms, e.g., personal and dispositional attribution (e.g., personal strength/self-perception and future perception), cohesion or family and social support [24], emotional stability, and social skills but not with cognitive intelligence [25]. Many studies have confirmed associations between resilience and such personality traits as openness to experience, extraversion, agreeableness, and conscientiousness and a negative link with neuroticism [26]. Many recent studies worldwide have investigated resilience as a protective factor in individuals particularly at risk of psychological distress and developing depression and anxiety symptoms (e.g., in the military, hospital staff) [27], as well as the role of resilience and meaning in life in dealing with the COVID-19 related stress, depressive symptoms, anxiety, panic, and behavioral disorders in daily life [22,28].

The findings of many studies have not only confirmed the link between resilience and other psychological variables but also considered resilience as a potential predictor of, e.g., locus of control [29] or a predictor of the quality of life in people with borderline personality disorder [30]. Results of most recent studies have shown that resilient individuals find meaning in life in difficult times more easily [5,22]. What is more, resilience and meaning in life were found to be significant predictors of posttraumatic growth [31]. The aforementioned results confirm that resilience should be considered as a resource facilitating the ability to create a better future and build meaning in life. 

### 1.2. Meaning in Life

There are three ways of understanding meaning in life (MIL) [32]. The first one is coherence—a sense of comprehensibility and sense made of life. The second is purpose—a sense of core goals, aims, and direction in life. The third is significance—a sense of life as an inherent value and having a life worth living [33]. These three dimensions of MIL are often seen as central. Although these three aspects of meaning are frequently treated by researchers as synonymous, they are potentially distinct. According to Heintzelman and King [34], purpose and significance are motivational components that are about the pursuit and attainment of worthwhile goals, while coherence is a cognitive component of MIL making sense of one’s experiences in life. Przepiórka [15] indicated that MIL “is determined by the individual’s personality structure, as well as by their personal goals and strivings” (p. 23). This is consistent with the definition proposed by Frankl [6], according to which MIL is a manifestation of values derived from creativity, experience, and attitude. MIL is rooted in personal resources. This author [6] also defined meaning in life as a life purpose and a life task. As an observer of life in the Nazi concentration camps during World War II, Frankl concluded that there were differences between those who managed to keep the sense of MIL and those who lost it during imprisonment. 

Previous studies have focused on MIL as a predictor of life satisfaction [35], psychological well-being [36,37], and stress [38,39]. MIL was found to mediate the relationship between optimism and subjective well-being [40], gratitude, grit, and suicide ideation [41]. Moreover, Hill et al. [42] found that the purpose in life is moderated by daily stress and well-being. However, factors that promote maintaining one’s sense of purpose in life despite hardship are also important. For example, Martela et al. [43] indicated that satisfaction of autonomy, competence, relatedness, and beneficence were predictors of MIL. Finding deeper meaning in one’s life can be supported by positive resources such as resilience, but also by finding connections between past and present events and expectations for the future [44]. 

### 1.3. Theoretical Conceptualization of Time Perspective

The tendency to focus on the past, present, or future is known as time perspective (TP) [45]. Time perspective is developed as life goes on, lending the framework of one’s personal experience covering the past, present, and future. TP is related to retaining events in memory, as well as to formulating goals or expectations, and it is rooted in a personal sense of coherence and continuity [46]. There are five theoretically orthogonal different time perspectives: Past Negative, Past Positive, Present Hedonistic, Present Fatalistic, and Future [45]. This multidimensional theory of time perspective affords a better understanding of an individual’s time perspective profile. Past Negative (PN) is manifested in a pessimistic attitude toward the past. Individuals with a high level of PN are likely to develop high levels of depression, anxiety, negative rumination, and low self-esteem [45]. PN is a maladaptive time orientation. PN is positively related to pessimism [47] and various negative aspects of health and coping, such as pain sensitivity and pain catastrophizing [48], catastrophizing as a coping mechanism [49], self-defeating humor [14], and mental health problems [50] and has an inverse relationship with resilience [10], openness to experience [51], positive affect, and sense of meaning in life [52]. Past Positive (PP) is a positive, glowing, and nostalgic construction of the past. PP was positively associated with well-being [52,53], affiliative humor, and self-enhancing humor [14] and was linked to spiritual growth, health-promoting lifestyle profile, and health responsibility in cardiac patients [54]. Present Fatalistic (PF) involves a fatalistic, helpless, and hopeless attitude to life and the future. PF was positively related to maladaptive variables such as negative affect [55] and negatively associated with general self-efficacy [55,56] and openness to experience [51] Present Hedonistic (PH) involves focusing on present enjoyment and pleasant activities. The positive consequences of PH favor well-being [57] and openness to experiences [49]. Future (FTP) is the orientation toward the achievement of future goals and planning. Individuals with a high level of FTP display a low level of ego-undercontrol, impulsive behavior, and sensation seeking. For example, FTP was positively correlated with resilience [11] and health responsibility [54]. Boyd and Zimbardo [58] claim that there should be a healthy balance between past, present, and future orientations. This allows an individual to properly adapt in the present and engage in goal-oriented behavior in the future. Time perspective allows a person to see how time frames influence one’s behavior and ability to adapt to change [46].

### 1.4. Moderating Effect of Time Perspective

Although previous research highlighted an indirect impact of time perspective on health and coping variables [49,59], to our knowledge, no studies to date have considered TP as a moderator between resilience and MIL. If TP is a moderator in the link between resilience and life meaning, the direction and intensity of this association would depend on the level of a specific TP type. Positive time appraisal refers to recalling and focusing on positive, pleasant events [60]. Negative time appraisal, on the other hand, is related to focusing and brooding on negative situations [55].

Resilience and its dimensions were found to be related to time perspective [10,13,14]. For example, optimism was positively linked with Past Positive, Present Positive, and Future Positive time perspectives and inversely related to Past Negative, Present Negative, and Future Negative time perspectives [12]. Previous research indicated that perceiving the past, present, and future as positive and focusing on the positive aspects of time may promote the perception that life is more meaningful and worth living [16,61]. In contrast, focusing on negative past events, perceiving the present as not favorable, and viewing the future with anxiety may lead to the experience of a lack of meaning in life [16,61]. These studies provide some evidence that TP may moderate the relationship between resilience and MIL. 

### 1.5. The Present Study: Hypotheses and Research Question

This study aimed to analyze the relationships between resilience, time perspective, and meaning in life. We assume that resilience can help one give meaning to his/her life and his/her life difficulties. Resilient individuals find MIL more easily, especially in difficult times [5,20]. Previous research has examined relationships between resilience and other psychological constructs [24,28], yet definitely fewer researchers have investigated the relationship between time perspective and other constructs [44,62]. Additionally, our aim was to verify whether time perspective can moderate the relationship between resilience and MIL. To the best of our knowledge, earlier studies failed to show how perception of one’s past, present, and future can modify the relationship between resilience and MIL.

However, previous research found associations between time perspective and resilience [10] and some factors describing resilience [12,13,14] and meaning in life [15,16]. More positive temporal orientations, namely Past Positive, Present Hedonistic, and Future, enhanced both resilience and MIL; more negative temporal perspectives, namely Past Negative and Present Fatalistic, led to low intensity of MIL [16], resilience [10], and optimism [12].

With regard to direct relationships, the following hypotheses were put forward:

**Hypothesis** **1** **(H1).** *Resilience will display a positive correlation with meaning in life.*

**Hypothesis** **2** **(H2).** *Past Negative (H2a) and Present Fatalistic (H2b) perspectives will be negatively associated with resilience, while Past Positive (H2c), Present Hedonistic (H2d), and Future (H2e) perspectives will be positively related to resilience.*

**Hypothesis** **3** **(H3).** *Past Negative (H3a) and Present Fatalistic (H3b) perspectives will be negatively related to meaning in life, while Past Positive (H3c), Present Hedonistic (H3d), and Future (H3e) perspectives will be positively related to meaning in life.*

With regard to moderated relationships, the following research question (RQ) was formed:

**RQ1.**  *Will time perspective moderate the relationship between resilience and meaning in life?*

## 2. Materials and Methods

### 2.1. Sampling and Data Collection 

A total of 363 adults (55% women), aged 18 to 70 (*M =* 28.23, *SD* = 11.3), took part in the study. A cross-sectional web-based survey was used to collect data. All respondents provided their informed consent online. The responses were anonymous, and confidentiality of information was assured. Participants were informed about the right to terminate the survey at any time they want. Data were collected between February and May 2021 using an online questionnaire distributed via social networking sites. A researcher was available by email, in case of any questions related to the process of completing the questionnaires. The data were saved on the web server of the first author of the study. A power analysis showed that the sample size is appropriate for the designed data analysis. 

Participants represented different Polish regions. They indicated their age, gender, level of education, professional activity, and place of living. Forty-six percent of respondents were employed, 44% were students, and 10% were unemployed. Forty percent of study participants lived in a city, 31% were town residents, and 29% lived in the country. The majority of respondents had completed secondary (56%) or higher (35%) education. The research was carried out online between February and May 2021. Participation was anonymous and voluntary. The invitation to participate in the study was disseminated on social networks. The study was conducted in compliance with ethical principles set out in the Declaration of Helsinki. All procedures performed in the study were in accordance with the ethical standards of the Scientific Research Ethics Committee of the Institute of Psychology, Pedagogical University of Krakow.

### 2.2. Measures

Resilience Measurement Scale SPP-25 [1] is a 25-item self-report questionnaire measuring five factors of resilience: perseverance and determination in action (e.g., “I never give up and I always fight for mine”), openness to new experience and sense of humor (e.g., “I am open to new experiences”), personal coping skills and tolerance of negative emotions (e.g., “In stressful situations, I concentrate and think clearly”), optimistic approach to life and the ability to focus in difficult situations (e.g., “I always have an optimistic outlook on life, regardless of the situation”), and tolerance of failures and treating life as a challenge (e.g., “In general, struggling with difficult situations strengthens me and develops me”). The responses are rated on a five-point scale ranging from 0 (“definitely not”) to 4 (“definitely yes”). This scale has good properties in assessing resilience in adults. In the current study, the internal consistency for total scale was α = 0.91, whereas reliability coefficients for particular subscales were respectively 0.77 for perseverance, 0.60 for openness, 0.75 for personal coping skills, 0.72 for optimistic approach, and 0.64 for tolerance of failures.

Meaning in life was measured using the Purpose in Life Questionnaire (PIL) [63]. We used the Polish short version consisting of 6 items [64]. In this measure, participants rate their answers on a seven-point Likert scale (e.g., “My existence is: 1—completely pointless, 7—purposeful and meaningful; I concluded that: 1—I have no purpose in life, 7—I have clear goals that give my full satisfaction”). The higher the result, the greater the meaning in life. This scale has good construct validity and good reliability (α = 0.90).

Time Perspective Inventory [45] consists of 56 items divided into 5 subscales: Past Negative scale (PN) assesses the tendency to focus on the negative past (e.g., “I think about bad things that have happened to me in the past”). Past Positive scale (PP) measures a positive assessment of the past. Tradition, history, and family are of high importance (e.g., “Thinking about the past gives me pleasure”). Present Fatalistic scale (PF) reflects an approach to the future and life which is fate-determined, full of helplessness and hopelessness with the belief that the future is out of control (e.g., “My life path is marked by forces over which I have no influence”). Present Hedonistic scale (PH) focuses on current pleasures, involving a careless approach to time and life (e.g., “It is important to make life exciting”); Future scale (FTP) reflects a future orientation, planning, and achievement of future goals (e.g., “I make to-do lists”). Respondents indicate their level of agreement with each of the statements on a 5-point scale (1 = “very untrue”, 5 = “very true”). Cronbach’s alpha value for the total ZTPI was 0.86; for the subscales, α = 0.84 for PN, 0.63 for PP, 0.73 for PF, 0.81 for PH, and 0.68 for FTP.

### 2.3. Statistical Analysis

IBM SPSS Statistics (version 26, PS IMAGO PRO 6.0, Predictive Solutions, Krakow, Poland) was used to calculate bivariate correlations. The serial moderation analysis included bootstrap analysis employing the PROCESS macro (MODEL 1,2) [65]. We conducted five separate moderation analyses with resilience as an independent variable and meaning in life as an output variable. Time perspective was the mediator. We estimated indirect effects using 5000 bootstrapped resamples at a 95% confidence interval. When the CI 95% did not cross zero, the indirect effects were significant.

## 3. Results

### 3.1. Pearson’s Correlation Coefficient

To explore the relationship between resilience, meaning in life, and time perspective, correlational analyses were performed. Table 1 shows intercorrelations (Pearson’s *r*) between analyzed variables. Resilience displayed a positive and significant correlation with meaning in life and Present Hedonistic and Future perspectives and an inverse correlation with Past Negative and Present Fatalistic perspectives. Past Negative and Present Fatalistic perspectives displayed a negative correlation with meaning in life, whereas Past Positive and Present Hedonistic perspectives displayed a positive correlation with meaning in life.

### 3.2. Moderating Effect 

**Figure 1:**
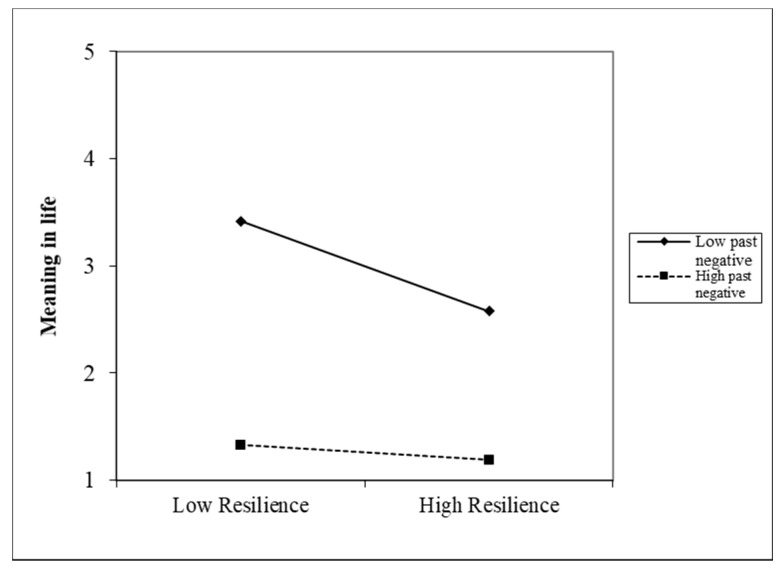
Graphical depiction of the moderation results with Past Negative perspective as a moderator.

**Figure 2:**
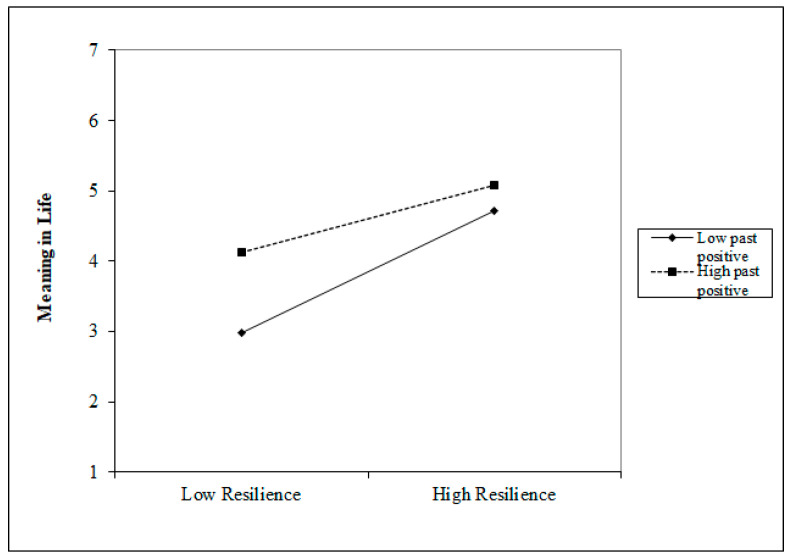
Graphical depiction of the moderation results with Past Positive perspective as a moderator.

We performed linear regressions to investigate the effects of resilience and specific time perspectives on meaning in life. In relation to RQ, resilience was an independent variable and meaning in life was a dependent variable in the used Hayes’s process [65]. We conducted five moderation analyses for each type of time perspective (Table 2). Past Negative and Past Positive perspectives significantly moderated the effect of resilience on meaning in life. A low level of Past Negative perspective was linked with a stronger positive relationship between resilience and meaning in life when compared with a high level of Past Negative perspective (Figure 1). Conversely, a high level of Past Positive perspective was linked with a stronger positive relationship between resilience and meaning in life when compared with a low level of Past Positive perspective (Figure 2).

Next, we conducted a moderation analysis with two moderators: Past Negative and Past Positive perspectives, with resilience as an independent variable and meaning of life as a dependent variable (process 2) [65]. The overall model explained 39% of the variance in MIL, and it was significant (F(348.5) = 44.931, *p* < 0.001). The resilience × PP and resilience × PN interactions were statistically significant, which means that PP and PN were moderators of the effect of resilience on MIL (Table 3).

Only when the Past Negative perspective is low and the Past Positive perspective is high, resilience has no impact on MIL. In the case of a low level of the Past Positive perspective and a high level of the Past Negative perspective, the relationship between resilience and MIL was significant and stronger than that in the case of other combinations of Past Positive and Past Negative perspective levels (see Table 4).

## 4. Discussion

The main goal of our study was to explore the relationship between resilience and meaning in life. We also examined the moderating role of time perspective in the relationship between resilience and meaning in life. The reported study is an attempt to integrate knowledge about the importance of temporal perspective in the context of enhancing and sustaining resources, using psychological resilience and meaning in life as examples. Having and sustaining positive personal resources is particularly important during the COVID-19 pandemic [4].

Just as we have expected, resilience was positively related to meaning in life. Higher resilience was associated with a stronger tendency to describe one’s life as meaningful. This result is consistent with the claim that personal resources are predictors of meaning in life [6,15]. Previous studies also pointed to the existence of a correlation between personality traits and meaning in life. For instance, gratitude and grit displayed a positive correlation with meaning in life in patients with suicide ideation [41]. Optimism was positively related to two ways of understanding meaning in life—presence of meaning in life and search for meaning in life—in elderly people [40], whereas Future time perspective displayed a positive correlation with MIL in emerging adults, young and middle-aged adults [66]. The outcomes might be interpreted in light of the Conservation of Resources theory [67], especially the construct of caravan passageways [68] where one resource reinforces and maintains another. In the case of our study, we can talk about mutual reinforcement of resilience and meaning in life. Hobfoll [68] emphasized that these resource passageways are disturbed during difficult, stressful situations, especially protracted ones like the COVID-19 pandemic. The results also support Frankl’s theory [6] of personality-based determinants of meaning in life. 

With regard to our second hypothesis, Negative Past and Present Fatalistic perspectives were inversely related to resilience. In contrast, Present Hedonistic and Future time perspectives were positively related to resilience. These outcomes are in line with previous findings confirming the existence of, among other things, a negative correlation between PN and resilience [10] and a positive relationship between resilience and FTP [11]. Our findings are also supported by previous outcomes which indicated Past Negative and Present Fatalistic perspectives as predictors of maladaptive functioning. For example, Past Negative orientation was related to catastrophizing [49], and Present Fatalistic perspective was related to alcohol abuse [59]. We have not observed any correlations between Past Positive perspective and resilience.

In line with our third hypothesis, Negative Past and Present Fatalistic perspectives were negatively associated with MIL; however, Past Positive and Present Hedonistic were positively related to MIL. Individuals who see their past as more positive, focus on the present time as pleasant, and focus less on feeling hopeless have a higher sense of MIL. Our results are partially consistent with the findings of Boniwell et al. [52] who found that Past Negative and Present Fatalistic perspectives showed an inverse correlation with purpose in life and that Past Positive and Future perspectives were positively related to purpose in life. Shterjovska and Achkovska-Leshkovska [16] found that presence of MIL was predicted by a high level of Past Positive, Present Hedonistic, and Future perspectives, and a low level of Past Negative orientations. In contrast, Baikeli et al. [66] found that Present Hedonistic perspective was not linked with MIL, but Future was positively related to MIL. The discrepancy in the results can be explained by the use of different tools to measure MIL. Baikeli et al. [66] used the Meaning in Life Questionnaire (MLQ) with two dimensions, namely the presence of MIL and the search for MIL, and the short version of the Zimbardo Time Perspective Inventory with only two perspectives—Present Hedonistic and Future. 

We also tested the moderating effect of time perspective on the relationship between resilience and meaning in life. The effect was confirmed only in the case of Past Positive and Past Negative time perspectives. In particular, resilience was less strongly related to the meaning in life in individuals who view their past as less positive and more negative, or as less positive and less negative. In only one case the moderating effect was not observed, i.e., when Positive Past perspective was high and Negative Past perspective was low. These results are consistent with the findings of Cunningham et al. [69] who revealed that the past perspective has a direct effect on the way people evaluate their life satisfaction. When evaluating the present and the future, the effect was indirect. In the case of the present perspective, the correlation with behaviors that affect the life circumstances was important for evaluating well-being, and in the case of the future perspective, the mediating role of actions promoting well-being was observed [69]. 

According to Zimbardo and Boyd [70], the current assessment of one’s situation, life, etc., is closely related to the construction of the past and to the anticipation of the future. Temporal cognitive frames are used to organize experiences; make meaning appropriately encoded and stored; and form expectations, goals, and perceptions. Indicating a temporal perspective allows for a change in an individual’s thought process and for cognitive restructuring of views about the past, present, and future [71]. The past is important for giving meaning to one’s life. The memory of negative experiences makes it difficult to give positive meaning to one’s life and, moreover, prevents one from using one’s resources. It can be said that it stops the movement of the caravan of passageways. 

On the other hand, a more positive past fosters the perception that life is worth engaging with and giving meaning to. The moderating role of past perspective suggests that in individuals with low Past Negative, the lower the resilience score, the higher the MIL, whereas in individuals with high Past Positive, the higher the resilience score, the higher the MIL. The present results provide further empirical evidence for the notion that a positive perception of the past is significant for current functioning [55,70]. Present time perspective (both Hedonistic and Fatalistic) and Future time perspective did not moderate the relationship between resilience and MIL.

Our findings are in line with the Construal-Level Theory of Psychological Distance [72]. The use of abstract constructs for psychologically distant events promotes effective functioning related to planning the future and understanding the world, oneself, and other people. Psychological distance from past events can make them a source of knowledge about one’s resources and a possibility to implement them at present and in the future, making life more meaningful. 

Taken together, our results are partially supported by the idea of the existence of optimal levels of time perspectives [52]. A balanced time perspective is described as a high level of Past Positive and Future perspectives, with a low level of Past Negative and Present Fatalistic perspectives [70]. Numerous studies have shown that the balanced time perspective is linked with greater flexibility and more effective responding in difficult situations [53]. An integrated past perspective enables a person to use his or her resources in order to make his or her life meaningful and orderly.

### Limitations

This study has certain limitations that have to be taken into account when interpreting the results. First, the main limitation of this study is the lack of a comparative sample; therefore, the results cannot be compared with the corresponding results from reference samples prior to the COVID-19 pandemic. Second, the sample was relatively small with respondents of highly varied age (range = 18–70 years). Third, as the cross-sectional design was used in the study, it is difficult to draw a conclusion about causality among the study variables. Finally, although measures used in this study were validated and widely used by other researchers, some bias may arise from the use of self-report methods. Therefore, it may be useful to monitor the tendency in social approval in future research. We consider this as another limitation of this study. 

## 5. Conclusions

Dealing with difficult, unexpected situations requires activation of internal resources in such a way as to be able to face them effectively while not suffering negative psychological effects. Among other things, maintaining the sense of meaning in life in demanding circumstances will translate into effective coping [39]. The present paper shows the relevance of positive resources in maintaining MIL. Resilience is a protective resource acting as a buffer against adverse effects of stress, but it also allows one to flourish and make one’s life meaningful [21,22]. Additionally, if the past appears positive, it gives one the strength to build resources in such a way that one can successfully deal with current situations. Thus, when providing psychological support, it is important to pay attention to the perception of the past as beneficial and understandable. Then, life will be filled with meaning and perceived as worth living. 

## Figures and Tables

**Table 1:** Descriptive statistics and the values of Pearson’s correlation coefficient for the relationship between resilience, meaning in life, and time perspectives.

	M (SD)	Meaning in Life	Past Negative	Past Positive	Present Fatalistic	Present Hedonistic	Future
Resilience	66.1 (13.4)	0.44 ***	−0.20 ***	0.02	−0.18 ***	0.18 ***	0.24 ***
Meaning in life	30.7 (8.5)		−0.38 ***	0.17 ***	−0.34 ***	0.17 ***	0.31
Past Negative	32.1 (7.7)			0.16 **	0.44 ***	0.16 **	0.14 **
Past Positive	30.6 (4.6)				0.26 **	0.24 ***	0.35 ***
Present Fatalistic	25.2 (5.7)					0.33 ***	−0.02
Present Hedonistic	54.5 (8.1)						0.05
Future	44.0 (5.8)						

Note: *** *p* < 0.001, ** *p* < 0.01.

**Table 2:** The results of analysis of the moderation effect of time perspective on resilience and meaning in life.

Moderator	R^2^	B	t	*p*	CI 95%		Interaction					
							B_L_	*p_L_*	B_M_	*p_M_*	B_H_	*p_H_*
Past Negative	0.31	0.35	3.56	<0.001	0.156	0.542	0.41	<0.001	0.60	<0.001	0.97	<0.001
Past Positive	0.28	−0.74	−5.37	<0.001	−1.02	−0.474	1.25	<0.001	0.83	<0.001	0.50	<0.001
Present Fatalistic	0.26	−0.03	−0.27	0.78	−0.281	0.211						
Present Hedonistic	0.20	−0.22	−1.38	0.16	−0.551	0.096						
Future	0.24	−0.19	−1.22	0.22	−0.509	0.118						

**Table 3:** Linear models of predictors of MIL.

	B	SE	t	*p*	LLCI	ULCI
Constant	5.2925	0.0497	106.5148	0.0000	5.1947	5.3902
Resilience	0.5562	0.0577	9.6421	0.0000	0.4427	0.6696
Past Positive	0.1787	0.0504	3.5478	0.0004	0.0796	0.2777
Resilience × PP	−0.2526	0.0484	−5.2148	0.0000	−0.3478	−0.1573
Past Negative	−0.3674	0.0509	−7.2235	0.0000	−0.4674	−0.2674
Resilience × PN	0.2050	0.0518	3.9603	0.0001	0.1032	0.3069

**Table 4:** The results of analysis of the moderation with two moderators for the independent variable resilience.

	B	t	*p*	CI 95%	
				Lower	Higher
Past Positive (low), Past Negative (low)	0.81	6.23	<0.001	0.555	1.06
Past Positive (low), Past Negative (medium)	1.10	9.58	<0.001	0.878	1.33
Past Positive (low), Past Negative (high)	1.39	9.73	<0.001	1.11	1.68
Past Positive (medium), Past Negative (low)	0.44	4.25	<0.001	0.236	0.642
Past Positive (medium), Past Negative (medium)	0.73	9.41	<0.001	0.579	0.886
Past Positive (medium), Past Negative (high)	1.02	9.19	<0.001	0.807	1.24
Past Positive (high), Past Negative (low)	0.13	1,18	0.238	−0.089	0.360
Past Positive (high), Past Negative (medium)	0.43	4.87	<0.001	0.255	0.601
Past Positive (high), Past Negative (high)	0.72	6.24	<0.001	0.494	0.949

## Data Availability

The dataset presented in this study is available on reasonable request from the corresponding author.

## References

[1] Ogińska-Bulik N., Juczyński Z. (2008). Skala pomiaru prężności SPP-25. [Resilience measurement scale SPP-25]. Nowiny Psychol..

[2] Connor K.M., Davidson J.R.T. (2003). Development of a new resilience scale: The Connor-Davidson Resilience Scale (CD-RISC). Depress. Anxiety.

[3] Wu G., Feder A., Cohen H., Kim J.J., Calderon S., Charney D.S., Mathé A.A. (2013). Understanding resilience. Front. Behav. Neurosci..

[4] Godor B.P., Van der Hallen R. (2021). Investigating the susceptibility to change of coping and resiliency during COVID-19. Scand. J. Psychol..

[5] Çolak T.S., Arıcı Özcan N., Peker A. (2021). The mediation role of personal meaning profile in the relationship between resilience capacity and meaning in life. Particip. Educ. Res..

[6] Frankl V.E. (1997). Man’s Search for Ultimate Meaning.

[7] Ostafin B.D., Proulx T. (2020). Meaning in life and resilience to stressors. Anxiety Stress Coping.

[8] Taylor S.E. (2003). Health Psychology.

[9] Helgeson V.S., Reynolds K.A., Tomich P.L. (2006). A meta-analytic review of benefit finding and growth. J. Consul. Clin. Psychol..

[10] Ge J., Yang J., Song J., Jiang G., Zheng Y. (2020). Dispositional mindfulness and past-negative time perspective: The differential mediation effects of resilience and inner peace in meditators and non-meditators. Psychol. Res. Behav. Manag..

[11] O’Neil E., Clarke P., Fido D., Vione K.C. (2020). The role of future time perspective, body awareness, and social connectedness in the relationship between self-efficacy and resilience. Int. J. Ment. Health Addict..

[12] Konowalczyk S., Buhl M., Moon J., Mello Z.R. (2019). The past, present, and future all matter: How time perspective is associated with optimism and sensation seeking among young adults. Res. Hum. Dev..

[13] Dwivedi A., Rastogi R. (2017). Proactive coping, time perspective and life satisfaction: A study on emerging adulthood. J. Health Manag..

[14] Hampes W. (2013). A pilot study of the relation between humor styles and the past-positive and past-negative time perspectives. Psychol. Rep..

[15] Przepiórka A. (2012). The relationship between attitude toward time and the presence of meaning in life. Int. J. Appl. Psychol..

[16] Shterjovska M., Achkovska-Leshkovska E. (2014). Time perspective as predictor of meaning in life. Int. J. Cogn. Res. Sci. Eng. Educ..

[17] Block J., Kremen A.M. (1996). IQ and ego-resiliency: Conceptual and empirical connections and separateness. J. Pers. Soc. Psychol..

[18] Luthar S.S., Cicchetti D., Becker B. (2000). The construct of resilience: A critical evaluation and guidelines for future work. Child. Dev..

[19] Robertson I., Cooper C.L., Sarkar M., Curran T. (2015). Resilience training in the workplace from 2003–2014: A systematic review. J. Occup. Organ. Psychol..

[20] Masten A.S., Cutuli J., Herbers J.E., Reed M.G., Lopez S.J., Snyder C.R. (2009). Resilience in Development. Oxford Handbook of Positive Psychology.

[21] Southwick S.M., Charney D.S. (2012). Resilience: The Science of Mastering Life’s Greatest Challenges.

[22] Yıldırım M., Arslan G., Aziz I.A. (2020). Why do people high in COVID-19 worry have more mental health disorders? The roles of resilience and meaning in life. Psychiatr. Danub..

[23] Leys C., Arnal C., Wollast R., Rolin H., Kotsou O., Fossion P. (2020). Perspectives on resilience: Personality trait or skill?. EJTD..

[24] Friborg O., Hjemdal O., Rosenvinge J.H., Martinussen M. (2003). A new rating scale for adult resilience: What are the central protective resources behind healthy adjustment?. Int. J. Methods Psychiatr. Res..

[25] Friborg O., Barlaug D., Martinussen M., Rosenvinge J.H., Hjemdal O. (2005). Resilience in relation to personality and intelligence. Int. J. Methods Psychiatr. Res..

[26] Oshio A., Taku K., Hirano M., Saeed G. (2018). Resilience and big five personality traits: A meta-analysis. Pers. Individ. Differ..

[27] Sefidan S., Pramstaller M., La Marca R., Wyss T., Sadeghi-Bahmani D., Annen H., Brand S. (2021). Resilience as a protective factor in basic military training, a longitudinal study of the Swiss Armed Forces. Int. J. Environ. Res. Public Health.

[28] Arslan G., Yıldırım M. (2020). Coronavirus Stress, Meaningful Living, Optimism, and Depressive Symptoms: A Study of Moderated Mediation Model. PsyArXiv.

[29] Kaplánová A., Gregor A. (2021). Self-acceptance, shame withdrawal tendencies and resilience as predictors of locus of control of behavior. Psychol. Stud..

[30] Guillén V., Tormo M.E., Fonseca-Baeza S., Botella C., Baños R., García-Palacios A., Jose Heliodoro Marco J.H. (2021). Resilience as a predictor of Quality of Life in participants with borderline personality disorder before and after treatment. BMC Psychiatry.

[31] Boullion G.Q., Pavlacic J.M., Schulenberg S.E., Buchanan E.M., Steger M.F. (2020). Meaning, social support, and resilience as predictors of posttraumatic growth: A study of the Louisiana flooding of August 2016. Am. J. Orthopsychiatry.

[32] Martela F., Steger M.F. (2016). The meaning of meaning in life: Coherence, purpose and significance as the three facets of meaning. J. Posit. Psychol..

[33] Heintzelman S.J., King L.A. (2014). Life is pretty meaningful. Am. Psychol..

[34] Heintzelman S.J., King L.A. (2014). (The feeling of) Meaning-as-information. Pers. Soc. Psychol. Rev..

[35] Bronk K.C., Hill P.L., Lapsley D.K., Talib T.L., Finch H. (2009). Purpose, hope, and life satisfaction in three age groups. J. Posit. Psychol..

[36] Fischer I.C., Secinti E., Cemalcilar Z., Rand K.L. (2021). Examining cross-cultural relationships between meaning in life and psychological well-being in Turkey and the United States. J. Happiness Stud..

[37] Yu E.A., Chang E.C. (2021). Relational meaning in life as a predictor of interpersonal well-being: A prospective analysis. Pers. Individ. Differ..

[38] Schnell T., Krampe H. (2020). Meaning in life and self-control buffer stress in times of COVID-19: Moderating and mediating effects with regard to mental distress. Front. Psychiatry.

[39] Park J., Baumeister R.F. (2016). Meaning in life and adjustment to daily stressors. J. Posit. Psychol..

[40] Ju H., Shin J.W., Kim C.W., Hyun M.H., Park J.W. (2013). Mediational effect of meaning in life on the relationship between optimism and well-being in community elderly. Arch. Gerontol. Geriatr..

[41] Kleiman E.M., Adams L.M., Kashdan T.B., Riskind J.H. (2013). Gratitude and grit indirectly reduce risk of suicidal ideations by enhancing meaning in life: Evidence for a mediated moderation model. J. Res. Pers..

[42] Hill P.L., Sin N.L., Turiano N.A., Burrow A.L., Almeida D.M. (2018). Sense of purpose moderates the associations between daily stressors and daily well-being. Ann. Behav. Med..

[43] Martela F., Ryan R.M., Steger M.F. (2018). Meaningfulness as satisfaction of autonomy, competence, relatedness, and beneficence: Comparing the four satisfactions and positive affect as predictors of meaning in life. J. Happiness Stud..

[44] Boniwell I. (2005). Beyond time management: How the latest research on time perspective and perceived time use can assist clients with time-related concerns. Int. J. Evid. Based Coach..

[45] Zimbardo P.G., Boyd J.N. (1999). Putting time in perspective: A valid, reliable individual-difference metric. J. Pers. Soc. Psychol..

[46] van Beek W., Berghuis H., Kerkhof A.J.F.M., Beekman A.T.F. (2011). Time perspective, personality and psychopathology: Zimbardo’s time perspective inventory in psychiatry. Time Soc..

[47] Shipp A.J., Edwards J.R., Lambert L.S. (2009). Conceptualization and measurement of temporal focus: The subjective experience of the past, present, and future. Organ. Behav. Hum. Decis. Process..

[48] Gacs A., Goertler S., Spasova S. (2020). Planned online language education versus crisis-prompted online language teaching: Lessons for the future. Forein Lang. Ann..

[49] Sobol-Kwapińska M., Plotek W., Bąbel P., Cybulski M., Kluzik A., Krystianc J., Mandecki M. (2017). Time perspective as a predictor of acute postsurgical pain and coping with pain following abdominal surgery. Eur. J. Pain.

[50] Laghi F., D’Alessio M., Pallini S., Baiocco R. (2009). Attachment representations and time perspective in adolescence. Soc. Indic. Res..

[51] Jochemczyk Ł., Pietrzak J., Buczkowski R., Stolarski M., Markiewicz Ł. (2017). You only live once: Present-hedonistic time perspective predicts risk propensity. Pers. Individ. Differ..

[52] Boniwell I., Osin E., Linley P.A., Ivanchenko G. (2010). A question of balance: Examining relationships between time perspective and measures of well-being in the British and Russian student samples. J. Posit. Psychol..

[53] Drake L., Duncan E., Sutherland F., Abernethy C., Henry C. (2008). Time perspective and correlates of wellbeing. Time Soc..

[54] Hamilton J.M., Kives K.D., Micevski V., Grace S.L. (2003). Time perspective and health promoting behavior in a cardiac rehabilitation population. J. Behav. Med..

[55] Stolarski M., Matthews G., Postek S., Zimbardo P.G., Bitner J. (2014). How we feel is a matter of time: Relationships between time perspectives and mood. J. Happiness Stud..

[56] Zebardast A., Besharat M.A., Hghighatgoo M. (2011). The relationship between self-efficacy and time perspective in students. Procedia Soc. Behav. Sci..

[57] Seema R., Sircova A. (2013). Mindfulness—A time perspective? Estonian Study. Balt. J. Psychol..

[58] Boyd J.N., Zimbardo P.G., Strathman A., Joireman J. (2005). Time perspective, health, and risk taking. Understanding Behavior in the Context of Time: Theory, Research, and Application.

[59] Wagner V., Acier D., Dietlin J.E. (2018). Mediation of time perspectives on inclinations to use alcohol and motivation to change relationship. J. Clin. Psychol..

[60] Szcześniak M., Timoszyk-Tomczak C. (2018). A time for being thankful: Balanced time perspective and gratitude. Stud. Psych..

[61] Steger M.F., Kashdan T.B., Sullivan B.A., Lorentz D. (2008). Understanding the search for meaning in life: Personality, cognitive style, and the dynamic between seeking and experiencing meaning. J. Pers..

[62] Przepiorka A., Sobol-Kwapinska M. (2021). People with positive time perspective are more grateful and happier: Gratitude mediates the relationship between time perspective and life satisfaction. J. Happiness Stud..

[63] Crumbaugh J.C., Maholick L.T. (1964). An experimental study in existentialism: The psychometric approach to Frankl’s concept of noogenic neurosis. J. Clin. Psychol..

[64] Życińska J., Januszek M. (2011). Test Sensu Życia (Purpose in Life Test, PIL) J.C. Crumbaugha i L.T. Maholicka: Analiza psychometryczna. Psychol. J..

[65] Hayes A.F. (2013). Introduction to Mediation, Moderation, and Conditional Process Analysis: A Regression-Based Approach.

[66] Baikeli R., Li D., Zhu L., Wang Z. (2021). The relationship between time perspective and meaning in life across different age stages in adulthood. Pers. Individ. Differ..

[67] Hobfoll S.E. (1989). Conservation of resources: A new attempt at conceptualizing stress. Am. Psychol..

[68] Hobfoll S.E. (2012). Conservation of resources and disaster in cultural context: The caravans and passageways for resources. Psychiatry.

[69] Cunningham K.F., Zhang J.W., Howell R.T., Stolarski M., Fieulaine N., van Beek W. (2015). Time perspective and subjective well-being. Time Perspective Theory; Review, Research and Application: Essays in Honor of Philip G. Zimbardo.

[70] Zimbardo P.G., Boyd J.N. (2008). The Time Paradox: The New Psychology of Time That Will Change your Life.

[71] Sword R.M., Sword R.K.M., Brunskill S.R., Stolarski M., Fieulaine N., van Beek W. (2015). Time perspective therapy: Transforming Zimbardo’s temporal theory into clinical practice. Time Perspective Theory; Review, Research and Application: Essays in Honor of Philip G. Zimbardo.

[72] Trop Y., Liberman N. (2010). Construal-level theory of psychological distance. Psychol. Rev..

